# Are Excessive Daytime Sleepiness and Lower Urinary Tract Symptoms the Triggering Link for Mental Imbalance? An Exploratory Post Hoc Analysis

**DOI:** 10.3390/jcm12226965

**Published:** 2023-11-07

**Authors:** Francesco Di Bello, Cristiano Scandurra, Benedetta Muzii, Claudia Colla’ Ruvolo, Gianluigi Califano, Edoardo Mocini, Massimiliano Creta, Luigi Napolitano, Simone Morra, Agostino Fraia, Vincenzo Bochicchio, Giovanni Salzano, Luigi Angelo Vaira, Francesco Mangiapia, Gaetano Motta, Giovanni Motta, Nelson Mauro Maldonato, Nicola Longo, Elena Cantone

**Affiliations:** 1 Department of Neurosciences, Reproductive Sciences and Odontostomatology, University of Naples “Federico II”, 80131 Naples, Italy; francesco.dibello@unina.it (F.D.B.); cristiano.scandurra@unina.it (C.S.); c.collaruvolo@gmail.com (C.C.R.); gianl.califano2@gmail.com (G.C.); max.creta@gmail.com (M.C.); luigi.napolitano@unina.it (L.N.); simone.morra@unina.it (S.M.); agostino.fraia@unina.it (A.F.); fmangiapia@gmail.com (F.M.); nelsonmauro.maldonato@unina.it (N.M.M.); nicola.longo@unina.it (N.L.); elena.cantone@unina.it (E.C.); 2 Department of Experimental Medicine, Sapienza University, 00185 Rome, Italy; edoardomocini@uniroma1.it; 3 Department of Humanistic Studies, University of Calabria, 87036 Rende, Italy; vincenzo.bochicchio@unical.it; 4 Maxillofacial Surgery Operative Unit, University Hospital of Naples “Federico II”, 80131 Naples, Italy; giovanni.salzano@unina.it; 5 Maxillofacial Surgery Operative Unit, Department of Medicine, Surgery and Pharmacy, University of Sassari, 07100 Sassari, Italy; lavaira@uniss.it; 6 ENT Unit, Department of Mental, Physical Health and Preventive Medicine, University of Campania “Luigi Vanvitelli”, 80131 Naples, Italy; gaetano.motta@unina2.it (G.M.); giovannimotta95@yahoo.com (G.M.)

**Keywords:** mental disorder, sleep apnea, sleep impairment, nocturia, lower urinary tract symptoms

## Abstract

Background: Both lower urinary tract symptoms (LUTS) and excessive daytime sleepiness (EDS) could negatively impair the patients’ quality of life, increasing the sensitivity to psychological distress that results in mental health disorders. The relationships of both urinary and respiratory domains with psychological distress in obstructive sleep apnea patients is still underestimated. Methods: This study was a post hoc analysis of a web-based Italian survey, which included 1998 participants. Three hierarchical multiple linear regression analyses with psychological distress as dependent variable were performed on the study of 1988 participants enrolled in the final analysis. Cohen’s f2 was used for the assessment of the effect size. Results: From the hierarchical multiple linear regression analyses, it emerged that the final statistical model (including sociodemographic characteristics, comorbidities, perceived urinary function, and excessive daytime sleepiness) for all dimensions accounted for 16.7% of the variance in psychological distress, with a medium effect size (f2 = 0.15). Conclusions: People reported psychological distress was impaired by the presence of LUTS and EDS. Specifically, our study showed that higher levels of distress were scored especially in young women exhibiting urinary symptoms and with high values of daytime sleepiness.

## 1. Introduction

Excessive daytime sleepiness (EDS) is defined as “the inability to stay awake and alert during the major waking episodes of the day, resulting in unintended lapses into drowsiness and sleep” [1,2]. Concerning EDS, lower urinary tract symptoms (LUTS) and, specifically, nocturia had a strong association with sleepiness [3,4]. Hence, LUTS had a negative impact on sleep efficiency, increasing daytime sleepiness [3]. Several comorbidities could be associated with these two realities, such as obstructive sleep apnea syndrome that has a hidden prevalence in the general population up to 50% [5,6]. Specifically, subjects are often unaware of the disorder, and the diagnosis may be performed after those symptoms occurred [5,6]. However, both EDS and LUTS lead to increased perceived psychological distress [7,8,9].

Distress is defined as a negative psycho-physical response to critical situations that breaks into the life of the individual, who experiences an imbalance between the magnitude of the event and the perceived resources available, leading to mental health endangering [10,11,12].

To cope with the stressors, individuals must bring into play capacities and resources that go beyond the normal repertoire, but this process may fail and give rise to complex symptoms such as mood disorders and anxiety [13,14,15]. Based on this conceptualization, Moos and Schaefer integrated the diagnosis and the course of a physical illness into critical experiences so that individuals with an organic pathology may implement massive efforts to make meaning out of this experience, as an attempt to develop appropriate coping strategies to deal with profound distress [9,10,11]. Difficulties related to the pathology, personal characteristics, and the social context in which the individual is embedded influence the distress and ways to cope with it [16]. Indeed, persisting distress can alter several physiological homeostasis, such as the activity of the autonomic nervous system [17].

Specifically, sleep disturbance (including EDS and/or sleep duration) plays a significant role in the assessment, pathogenesis, and treatment of mood disorders and related-stress [18]. People affected by LUTS may tend to be more anxious, to have difficulty concentrating and may be more prone to depression [19]. Furthermore, both LUTS and EDS could reduce the subjects’ productivity, becoming an impediment to daily actions [20]. As a result, both LUTS and EDS could negatively impair the patients’ quality of life (QoL), increasing the sensitivity to psychological distress that results in mental health disorders [3,7,21,22,23]. To the best of our knowledge, no previous author had investigated the relationships of both urinary and respiratory domains with psychological distress. The aim of the current study was to assess the burden of urinary symptoms and EDS on psychological distress in Italian participants in an online survey.

## 2. Materials and Methods

### 2.1. Participants

This study was a post hoc analysis of a web-based Italian survey, which included 1998 participants. Firstly, the survey was administered via Google Forms between 17 July and 31 October 2022 [24]. The participants were reached through advertisements published on online social media (Instagram, Facebook and Whatsapp), sharing their experiences about being potentially affected by obstructive sleep apnea syndrome (OSAS). In addition, a snowball recruitment procedure was performed, by asking people interested in the study to share the survey link to other potential interested participants they personally knew. All participants took part voluntarily in our survey without any economic incentive for their participation. Through the link shared on social media platforms, participants were directed to the informed consent of the study on the Google Forms first page. Thus, after reading the information regarding the objectives, benefits, risks, information about researchers, and anonymity of the survey, the participant had to click “I accept to participate in the study” to skip forward. To avoid missing data, all questions were mandatory, but participants were informed about their right to stop the survey at any moment they wanted. The study was conducted in accordance with the EU General Data Protection Regulation and the Declaration of Helsinki on Ethical Principles for Medical Research Involving Human Subjects.

Participants could be enrolled in the survey if they satisfied the following inclusion criteria: (1) being at least 18 years old (the Italian age of consent); (2) having experienced snoring or apnea during the sleeping, with a suspicious diagnosis of OSAS; (3) having experienced excessive daytime sleepiness, with a suspicious diagnosis of OSAS; and (4) not having experienced snoring or apnea during the sleeping but having been screened for OSAS. A total of 1998 participants took part in the survey. Among these, 10 did not satisfy at least one of the inclusion criteria. Thus, the final sample was composed of 1988 Italian participants.

### 2.2. Measures

#### 2.2.1. Demographics and Clinical Information

The following sociodemographic and clinic variables were collected: age, gender (men vs. women), weight, height, body mass index (BMI), comorbidities (e.g., diabetes, respiratory disease [including asthma, chronic obstructive pulmonary disease], dysthyroidism, hypertension; yes vs. no), and smoking history (yes for “past and current smokers” vs. no for “never smoker”).

#### 2.2.2. Psychological Distress

Psychological distress was evaluated with the Kessler Psychological Distress Scale (K-10) [25], a 10-item questionnaire detecting psychological distress based on questions about anxiety and depressive symptoms experienced during the last 30 days. The answer options ranged from “1” (none of the time) to “5” (all the time). Higher scores indicate greater psychological distress. Consistently with Andrews and Slade [26], the cut-off scores adopted of <20, 20–24, 25–29, ≥30 were set to detect the likelihood of absent, mild, moderate, and severe psychological distress, respectively. Moreover, the questionnaire achieved the ability to discriminate DSM-IV cases from non-cases [25]. The α coefficient for the current sample was 0.93.

#### 2.2.3. Excessive Daytime Sleepiness

The subjective perceptions of EDS were measured through the Epworth Sleepiness Scale (ESS) [27]. The ESS represents a subjective measure of a patient’s sleepiness related to a respiratory impairment [28]. The test explores 8 situations in which the respondents rate the tendency to become sleepy on a scale ranging from “0” (no chance of dozing) to “3” (high chance of dozing). Specifically, the tendency to become sleepy is explored during “sitting and reading”, “watching TV”, “sitting inactive in a public place”, “as a passenger in a car for an hour without a break”, “lying down to rest in the afternoon when circumstances permit”, “sitting and talking to someone”, “sitting quietly after a lunch without alcohol” and “in a car, while stopped for a few minutes in traffic”. A total score <6 is unlikely to be abnormally sleepy, while scores of 6–10, 11–12, 13–15, and 16–24 are associated with a higher normal, mild excessive, moderate excessive, and severe excessive amount of daytime sleepiness, respectively, seeking medical attention [27]. The α coefficient for the current sample was 0.81.

#### 2.2.4. Urinary Functioning

The urinary functioning was measured through the validated International Prostate Symptom Score (IPSS) questionnaire. The IPSS is a 7-item questionnaire, plus 1 question relating to the overall QoL, assessing the severity of LUTS [29,30]. It could be used in both genders due to the low sex- and disease-specificity, achieving the same psychometric properties [31,32]. The items investigated the presence of incomplete emptying, frequency, intermittency, urgency, weak stream, straining, and nocturia. Each item admits a 5-point answer, from “0” (Not at all) to “5” (Almost always). The IPSS score results from the sum of the ordinal responses to the 7 items. Specifically, the patients result mildly, moderately, or severely symptomatic when the total scores are 0–7, 8–19 or 20–35, respectively. The overall QoL due to urinary symptoms, instead, admit 6-points answer to “0” (Delighted) to “6” (Terrible). The α coefficient for the current sample was 0.80.

#### 2.2.5. Statistical Analyses

We first analyzed participants’ characteristics through descriptive statistics and bivariate correlations between study variables with Pearson’s coefficient. Then, we performed three hierarchical multiple linear regression analyses with psychological distress as dependent variable. In the tested models we entered age, gender, and BMI as covariates in step 1, smoking and comorbidities in step 2, and daytime sleepiness and urinary symptoms in step 3. Cohen’s f2 was used for the assessment of the effect size, with f2 ≥ 0.02, f2 ≥ 0.15, and f2 ≥ 0.35 representing respectively small, moderate, and large effect sizes [33]. Furthermore, to avoid multicollinearity variance inflation factor (VIF) and Durbin–Watson’s test were run. Conventionally, VIFs above 5 are acceptable, while Durbin–Watson’s index near 2 is optimal [34]. All statistical analyses were performed using the Statistical Package for the Social Sciences (SPSS) version 28, setting the significance level for all statistical tests at α = 0.05.

## 3. Results

### 3.1. Socio-Demographic and Clinical Characteristics of Participants

Full socio-demographic and clinical characteristics of 1988 participants are reported in Table 1. Within the overall participants, the mean age was 34.11 ± 10.45. According to the gender, 238 (12%) and 1750 (88%) were men and women, respectively. The overall mean BMI value was 26.20 ± 21.17. According to the presence of comorbidities, 44 (2.2%), 91 (4.6%), 207 (10.4%), and 99 (5.0%) patients were affected by diabetes, respiratory disease, dysthyroidism, and arterial hypertension, respectively. Furthermore, 429 (21.6%) had smoking habits. From the K-10 questionnaire, it emerged that 395 (19.9%), 373 (18.8%), and 610 (30.7%) participants harbored the likelihood of mild, moderate, and severe psychological distress, respectively. According to the ESS evaluation, 1025 (51.6%) and 666 (33.5%), 142 (7.1%), 93 (4.7%), and 62 (3.1%) participants harbored low sleepiness and higher normal, mild excessive, moderate excessive, severe excessive daytime sleepiness, respectively. According to the IPSS questionnaire, 1144 (57.5%), 739 (37.2%) and 105 (5.3%) participants harbored mild, moderate, and severe group categories, respectively. Finally, 872 (43.9%), 392 (19.7%), 352 (17.7%), 229 (15.5%), 96 (4.8%) and 47 (2.4%) had a LUTS-QoL defined as “delighted”, “pleased”, “mostly satisfied”, “mixed”, “mostly dissatisfied”, “unhappy” and “terrible”, respectively.

### 3.2. Bivariate Correlations

Pearson correlations (reported in Table 2) indicated that the majority of all socio demographics characteristics and clinical variables correlated positively with each other, except for hypertension that is negatively correlated with being a woman. Additionally, psychological distress is correlated positively with being a woman and dysthyroidism correlated negatively with age. Finally, perceived urinary functioning and EDS are correlated positively with each other and with psychological distress.

### 3.3. Associations of Perceived Urinary and Respiratory Functioning with Psychological Distress

A hierarchical multiple linear regression analysis was performed to assess the associations of perceived urinary and respiratory functioning with psychological distress in the survey participants (Table 3). Socio-demographic characteristics in model 1 explained 3.7% of variation in psychological distress, with a medium effect size (f2 = 0.20). Specifically, being women and younger were associated with higher scores on psychological distress. Adding clinical variables (such as smoke habits and comorbidities) in model 2 of the regression model explained a significant additional of 0.6% of the variation in psychological distress, but none of the clinical variables resulted statistically significant. Indeed, only women and younger age were statistically significantly associated with higher scores on psychological distress. Finally, adding perceived urinary functioning and EDS in model 3 of the regression model explained a significant additional 12.5% of the variation in psychological distress. Specifically, dysthyroidism, perceived urinary functioning and EDS were associated with higher scores on psychological distress in the model 3, with the constant influence of gender and age. The final statistical model for all dimensions accounted for 16.7% of the variance in psychological distress, with a medium effect size (f2 = 0.13).

## 4. Discussion

The current study was aimed at assessing the association between urinary symptoms, EDS, and psychological distress in web-based Italian survey participants. Results mainly indicated that, among the domains analyzed, the impairment of urinary functioning and EDS were moderately associated with psychological distress, with a constant influence of age and gender. In addition, the number of participants hired is noteworthy and shows a warning signal about the described conditions that cannot be ignored.

Firstly, we observed that there was a wider participation at survey of women (88% vs. 12% of men; ratio 8:1). Specifically, the overall participants were young (with a mean age of 34.11 ± 10.45), overweight (mean BMI of 26.20 ± 21.17), and smokers (nearly one-third). According to their medical history, participants are generally healthy with less than half affected by the most prevalent comorbidities. Specifically, 2.2%, 4.6%, 5%, 10.4% of participants were affected by diabetes, respiratory disease, arterial hypertension, and dysthyroidism, respectively. As to the significance of covariate variables, we found that being young and woman was associated with higher scores on psychological distress. We underlined that the survey took place in the post COVID-19 era, reflecting its consequences. Indeed, during the COVID-19 pandemic the feeling of anxiety and depression increased exponentially, specifically in the sub-population of young adults, such as college students [35,36]. Consistent with our data, a longitudinal study conducted in the UK revealed that psychological distress was higher than expected particularly among people aged 18–34 years [37]. Furthermore, it is well known that women are more prone to seek medical care compared to men, even with the same physical or psychiatric problems [38]. Specifically, women having psychosomatic problems experienced more intense, frequent, and numerous symptoms, with respect to men [39].

Secondly, ESS and IPSS questionnaires resulted in noteworthy results, especially proportionate to the participants’ number. Indeed, 15% of participants and almost a half of the total sample harbored excessive daytime sleepiness and moderate-to-severe urinary symptoms, respectively. Furthermore, the K-10 results were alarming with one-half of participants harboring highest scores (one third with value over 30), underpinning a likelihood to have a moderate-to-severe mental disorder. A significant correlation between the psychological distress and urinary alterations (r = 0.32, *p* < 0.001), and the psychological distress and EDS (r = 0.22, *p* < 0.001), emerged. Furthermore, the respiratory impairment and urinary alterations were also significantly correlated with each other (r = 0.17, *p* < 0.001). These findings reflected that the presence of both urinary impairment and EDS influences the stress of participants, regardless of the severity of symptoms experienced. Moreover, we identified that the magnitude of psychological distress may be driven by the women, but the addition of comorbidities, and urinary and respiratory symptoms to the hierarchical regression model seemed to flatten this phenomenon, confirming the results achieved. However, a possible explanation for the strong correlation between urinary impairment and EDS is related to sleeping disruption. Indeed, among LUTS, nocturia, defined as “waking at night to void”, has a prevalence rate of 54.5% and 48.6% for females and males, respectively, in 18 years or older people [40,41,42]. Therefore, nocturia may contribute to reducing nighttime sleeping, increasing EDS and stress-related EDS. However, further longitudinal studies are required to address these speculative hypothesis.

To the best of our knowledge, no previous study had investigated the relationships of both urinary and respiratory domains with psychological distress. Indeed, Bai et al. studied the presence of psychological distress with the Beck Anxiety Inventory and Perceived Stress Scale in chronic prostatitis/chronic pelvic pain syndrome patients with LUTS [43]. Specifically, they identified a strong correlation between LUTS and severity of psychological stress [43]. Moreover, Guglielmi et al. analyzed in truck drivers the relationship between sleep problems and mental health, scored with a general health questionnaire [9]. The main results suggested that the high prevalence of sleep problems and low quality of sleep can worsen the general and psychological well-being of the workers. In our report, the K-10 scale was an accurate instrument to identify the presence of psychological stress regardless of the symptom’s severity. The main results of the current study consist in the fact that the impaired urinary functioning and EDS were associated with higher scores on psychological distress among the models hypothesized. The final statistical model for all dimensions accounted for 16.7% of the variance in psychological distress, with a medium effect size (f2 = 0.15). Altogether, the co-presence of urinary dysfunction and EDS in patients must be attentive by physicians for the risk of mental health consequences [44,45]. Indeed, Coyne et al. assessed the risk of a greater mental health burden in individuals with urgency urinary incontinence (UI) combined with stress UI or other incontinence [46]. Furthermore, the observations of Zuluaga et al. from the COBaLT study were consistent with previous authors in emphasizing the risk of developing depression in patients’ with severe LUTS [9,45,47]. Therefore, it seems that the impact on mental health and QoL must be ascribable to the complex relationship between urinary symptoms, EDS, and the modality in which the individual perceives and deals with psychological distress.

Our study is not devoid of limitations. First, the cross-sectional nature of the study allowed for a picture of the sample only, taken as a representative of a larger population. Moreover, the survey was designed as not piloted to avoid the missing of important data, useful to understand the OSAS complexity. Future research should correct this aspect, considering a wider sample size and evaluating variable relationships in a longitudinal manner (e.g., by assessing the severity of symptoms and the impact on psychological distress or the depression syndrome occurred). Second, there is a clear gender disparity in the sample, as 1750 participants (88%) were female, and only 238 (12%) were male. Moreover, only Italian participants were recruited. Future research should consider whether including a more gender- and ethnical-balanced sample might offer the opportunity to improve these results. Third, the ESS questionnaire represents a subjective measure of a patient’s sleepiness that could be influenced by several additional factors. Therefore, future studies could include a more objective measure to evaluate DS, improving the accuracy levels of the measurement. Fourth, this study was conducted online, thus only participants who have internet access were recruited. As a consequence, elderly people or potential participants with disadvantaged socio-economic conditions were ruled out. Thus, future studies could include a wider sample, guarantying a wider access to the study participation.

Despite limitations, the presence of such a wide participation endorsed that the current findings may improve the management of patients experiencing LUTS and EDS. Indeed, it is important to stress that clinicians (psychologists and/or psychiatrists) must be aware that urinary and respiratory impairments could be a triggering factor for mental distress and could increase the psychological distress leading to mental disorders such as depression and anxiety. Conversely, for the urologists and ENT specialists, an appropriate patients’ counseling (using K-10 tool as screening questionnaire for distress) and a clinical joint assessment are required to better improve the awareness of patients on the symptoms they experience. Finally, LUTS and EDS could also worsen these sudden conditions when present. This is the reason it is fundamental that clinicians would offer an accurate diagnostic therapeutic management for the underlying conditions and psychological support for relieving the stress of urinary and respiratory dysfunctions.

## 5. Conclusions

People reported that their psychological distress was impaired by the presence of LUTS and EDS. Specifically, our study showed that higher levels of distress were scored, especially in young women exhibiting urinary symptoms and with a high value of daytime sleepiness. These aspects must be considered in the clinical management of these subsets of patients to improve their QoL and to reduce the mental health burden.

## Figures and Tables

**Table 1:** Descriptive statistics of 1988 subjects enrolled through cross-sectional web-based Italian survey, administered via Google Forms, between 17 July and 31 October 2022.

	Overall N = 1988
Age (M ± SD)	34.11 ± 10.45
Gender n (%)	
Men	238 (12)
Women	1750 (88)
BMI (M ± SD)	26.20 ± 21.17
Diabetes	
No	1944 (97.8)
Yes	44 (2.2)
Respiratory disease	
No	1897 (95.4)
Yes	91 (4.6)
Dysthyroidism	
No	1781 (89.6)
Yes	207 (10.4)
Arterial hypertension	
No	1889 (95.0)
Yes	99 (5.0)
Smoking habits	
No	1559 (78.4)
Yes	429 (21.6)
K-10 cut-off, n (%)	
Likely to be well (<20)	610 (30.7)
Likely to have a mild well (20–24)	395 (19.9)
Likely to have a moderate well (25–29)	373 (18.8)
Likely to have a severe well (≥30)	610 (30.7)
Epworth sleepiness scale cut-off, n (%)	
Low sleepiness (<6)	1025 (51.6)
Higher normal daytime sleepiness (6–10)	666 (33.5)
Mild excessive daytime sleepiness (11–12)	142 (7.1)
Moderate excessive daytime sleepiness (13–15)	93 (4.7)
Severe excessive daytime sleepines (16–24)	62 (3.1)
International Prostate Symptoms Score cut-off, n (%)	
Mild (0–7)	1144 (57.5)
Moderate (8–19)	739 (37.2)
Severe (20–35)	105 (5.3)
QoL cut-off, n (%)	
Delighted (0)	872 (43.9)
Pleased (1)	392 (19.7)
Mostly satisfied (2)	352 (17.7)
Mixed: Equally satisfied/dissatisfied (3)	229 (15.5)
Mostly dissatisfied (4)	96 (4.8)
Unhappy (5)	47 (2.4)
Terrible (6)	0 (0)

Abbreviation: BMI = body mass index; K-10 = Kessler Psychological Distress Scale; M = mean; QoL = quality of life; SD = standard deviation.

**Table 2:** Bivariate correlations between psychological distress, clinical characteristics, and K-10, ESS and IPSS scores.

	Age	Gender	BMI	K-10	ESS	IPSS	Smoking Habits
Age	-						
Gender	−0.035	-					
BMI	0.082 **	0.003	-				
K-10	−0.179 **	0.074 **	0.029	-			
ESS	0.013	−0.028	−0.011	0.224 **	-		
IPSS	−0.035	0.076 **	0.032	0.324 **	0.173 **	-	
Smoking habits	−0.10 **	−0.09 **	−0.04	0.04	0.04	0.03	-

** *p* value < 0.001; Abbreviation: BMI = body mass index; ESS = Epworth sleepiness scale; IPSS = International Prostate Symptoms Score; K-10 = Kessler Psychological Distress Scale.

**Table 3:** Hierarchical multiple linear regression of psychological distress on urinary and respiratory functioning.

	Psychological Distress		
	B (SE)	*β*	95% CI
Model 1—Sociodemographics			
Age	−0.14 (0.01)	−0.181 **	−0.184, −0.113
Gender	1.79 (0.58)	3.082 **	0.65, 2.93
BMI	0.02 (0.01)	1.95	0.00, 0.03
R^2^ = 0.037; F = 26.61 **			
Model 2—Comorbidities			
Age	−0.16 (0.02)	−0.196 **	−0.199, −0123
Gender	1.74 (0.59)	0.07 *	0.59, 2.90
BMI	0.01 (0.01)	0.036	−0.003, 0.03
Smoking habits	0.70 (0.46)	0.034	−0.20, 1.61
Diabetes	1.66 (1.30)	0.03	−0.87, 4.20
Respiratory disease	−0.16 (0.90)	−0.004	−1.94, 1.60
Dysthyroidism	2.01 (0.62)	0.07	0.78, 3.24
Arterial hypertension	1.15 (0.93)	0.03	−0.67, 2.98
R^2^ = 0.043; ΔR^2^ = 0.008; F = 3.38 *			
Model 3—Respiratory and urinary functioning			
Age	−0.15 (0.01)	−0.18 **	−0.19, −0.11
Gender	1.28 (0.55)	0.05 *	0.20, 2.37
BMI	0.01 (0.01)	0.03	−0.01, 0.02
Smoking habits	0.38 (0.43)	0.02	−0.47, 1.22
Dyabetes	1.54 (1.20)	0.03	−0.82, 3.91
Respiratory disease	−0.23 (0.84)	−0,01	−1.88, 1.42
Dysthyroidism	1.58 (0.58)	0.05 *	0.43, 2.72
Arterial hypertension	0.53 (0.87)	0.01	−1.17, 2.24
ESS	0.36 (0.04)	0.17 **	0.27, 0.44
IPSS	0.41 (0.03)	0.28 **	0.35, 0.47
R^2^ = 0.167; ΔR^2^ = 0.125; F = 148.94 **			

Notes. B = Unstandardized regression coefficient; SE = Standard error; CI = Confidence interval; β = Unstandardized regression coefficient; F = Explained variance R^2^ = R-square; ΔR^2^ = Change in R^2^; ** *p* < 0.001; * *p* < 0.05. Abbreviation: BMI = body mass index; ESS = Epworth sleepiness scale; IPSS = International Prostate Symptoms Score; K-10 = Kessler Psychological Distress Scale.

## Data Availability

The data and materials that support the findings of this study are available from the corresponding author upon reasonable request.

## References

[1] (2004). The International Classification of Sleep Disorders Revised 1997. Sleep Medicine Secrets.

[2] Morrison I., Riha R.L. (2012). Excessive daytime sleepiness and narcolepsy—An approach to investigation and management. Eur. J. Intern. Med..

[3] Martin S.A., Appleton S.L., Adams R.J., Taylor A.W., Catcheside P.G., Vakulin A., McEvoy R.D., Antic N.A., Wittert G.A. (2016). Nocturia, Other Lower Urinary Tract Symptoms and Sleep Dysfunction in a Community-Dwelling Cohort of Men. Urology.

[4] Di Bello F., Napolitano L., Abate M., Collà Ruvolo C., Morra S., Califano G., Capece M., Creta M., Scandurra C., Muzii B. (2023). Nocturia and obstructive sleep apnea syndrome: A systematic review. Sleep Med. Rev..

[5] Maniaci A., Iannella G., Cocuzza S., Vicini C., Magliulo G., Ferlito S., Cammaroto G., Meccariello G., De Vito A., Nicolai A. (2021). Oxidative Stress and Inflammation Biomarker Expression in Obstructive Sleep Apnea Patients. J. Clin. Med..

[6] Bang S., Tukhtaev S., Ko K.J., Han D.H., Baek M., Jeon H.G., Cho B.H., Lee K.-S. (2022). Feasibility of a deep learning-based diagnostic platform to evaluate lower urinary tract disorders in men using simple uroflowmetry. Investig. Clin. Urol..

[7] Braga A.A.N.M., Veiga M.L.T., Ferreira M.G.C.d.S., Santana H.M., Barroso U. (2019). Association between stress and lower urinary tract symptoms in children and adolescents. Int. Braz. J. Urol..

[8] LaGrotte C., Fernandez-Mendoza J., Calhoun S.L., Liao D., Bixler E.O., Vgontzas A.N. (2016). The relative association of obstructive sleep apnea, obesity and excessive daytime sleepiness with incident depression: A longitudinal, population-based study. Int. J. Obes..

[9] Guglielmi O., Magnavita N., Garbarino S. (2018). Sleep quality, obstructive sleep apnea, and psychological distress in truck drivers: A cross-sectional study. Soc. Psychiatry Psychiatr. Epidemiol..

[10] Bochicchio V., Mezzalira S., Maldonato N.M., Cantone E., Scandurra C. (2023). Olfactory-related quality of life impacts psychological distress in people with COVID-19: The affective implications of olfactory dysfunctions. J. Affect. Disord..

[11] Scandurra C., Zapparella R., Policastro M., Continisio G.I., Ammendola A., Bochicchio V., Maldonato N.M., Locci M. (2022). Obstetric violence in a group of Italian women: Socio-demographic predictors and effects on mental health. Cult. Health Sex..

[12] Gasparro R., Scandurra C., Maldonato N.M., Dolce P., Bochicchio V., Valletta A., Sammartino G., Sammartino P., Mariniello M., di Lauro A.E. (2020). Perceived Job Insecurity and Depressive Symptoms among Italian Dentists: The Moderating Role of Fear of COVID-19. Int. J. Environ. Res. Public Health.

[13] Schneiderman N., Ironson G., Siegel S.D. (2005). Stress and Health: Psychological, Behavioral, and Biological Determinants. Annu. Rev. Clin. Psychol..

[14] Holahan C.J., Moos R.H. (1986). Personality, coping, and family resources in stress resistance: A longitudinal analysis. J. Personal. Soc. Psychol..

[15] Schaefer J.A., Moos R.H. (1996). Effects of work stressors and work climate on long-term care staff’s job morale and functioning. Res. Nurs. Health.

[16] Scandurra C., Muzii B., La Rocca R., Di Bello F., Bottone M., Califano G., Longo N., Maldonato N.M., Mangiapia F. (2022). Social Support Mediates the Relationship between Body Image Distress and Depressive Symptoms in Prostate Cancer Patients. Int. J. Environ. Res. Public Health.

[17] O’Connor D.B., Thayer J.F., Vedhara K. (2021). Stress and Health: A Review of Psychobiological Processes. Annu. Rev. Psychol..

[18] Reynolds C.F. (2011). Troubled Sleep, troubled minds, and DSM-5. Arch. Gen. Psychiatry.

[19] Vrijens D., Drossaerts J., van Koeveringe G., Van Kerrebroeck P., van Os J., Leue C. (2015). Affective symptoms and the overactive bladder—A systematic review. J. Psychosom. Res..

[20] Sathya K., Sri Sakthi D., Jayakumar N.D., Meignana A.I. (2023). Sleep disorders and work-related stress with oral hygiene among Indian shift workers. Bioinformation.

[21] Creta M., Sagnelli C., Celentano G., Napolitano L., La Rocca R., Capece M., Califano G., Calogero A., Sica A., Mangiapia F. (2021). SARS-CoV-2 infection affects the lower urinary tract and male genital system: A systematic review. J. Med. Virol..

[22] Fusco F., Palmieri A., Ficarra V., Giannarini G., Novara G., Longo N., Verze P., Creta M., Mirone V. (2016). α1-Blockers Improve Benign Prostatic Obstruction in Men with Lower Urinary Tract Symptoms: A Systematic Review and Meta-analysis of Urodynamic Studies. Eur. Urol..

[23] Verze P., Califano G., Sokolakis I., Russo G.I., Hatzichristodoulou G., Musi G., Creta M., EAU-YAU Men’s health working party (2019). The impact of surgery for lower urinary tract symptoms/benign prostatic enlargement on both erectile and ejaculatory function: A systematic review. Int. J. Impot. Res..

[24] Google Docs OSAS, Disturbi Urinari e Sessuali: Survey Italiana. https://docs.google.com/forms/d/e/1FAIpQLScWigwsHhvQ2S8SDjke35yyCcoDHAy0868s5Gosyw6bC-mGTA/viewform?ts=62d540ee&usp=embed_facebook.

[25] Kessler R.C., Andrews G., Colpe L.J., Hiripi E., Mroczek D.K., Normand S.-L.T., Walters E.E., Zaslavsky A.M. (2002). Short screening scales to monitor population prevalences and trends in non-specific psychological distress. Psychol. Med..

[26] Andrews G., Slade T. (2001). Interpreting scores on the Kessler Psychological Distress Scale (K10). Aust. N. Z. J. Public Health.

[27] Johns M.W. (1991). A New Method for Measuring Daytime Sleepiness: The Epworth Sleepiness Scale. Sleep.

[28] Fabbri M., Beracci A., Martoni M., Meneo D., Tonetti L., Natale V. (2021). Measuring Subjective Sleep Quality: A Review. Int. J. Environ. Res. Public Health.

[29] Uroweb—European Association of Urology EAU Guidelines on Non-Neurogenic Female LUTS—INTRODUCTION—Uroweb. https://uroweb.org/guidelines/non-neurogenic-female-luts.

[30] Uroweb—European Association of Urology EAU Guidelines on the Management of Non-Neurogenic Male LUTS—INTRODUCTION—Uroweb. https://uroweb.org/guidelines/management-of-non-neurogenic-male-luts.

[31] Zhang H.L., Huang Z.G., Qiu Y., Cheng X., Zou X.Q., Liu T.T. (2017). Tamsulosin for treatment of lower urinary tract symptoms in women: A systematic review and meta-analysis. Int. J. Impot. Res..

[32] Okamura K., Nojiri Y., Osuga Y., Tange C. (2009). Psychometric Analysis of International Prostate Symptom Score for Female Lower Urinary Tract Symptoms. Urology.

[33] Cohen (1988). Statistical Power Analysis for the Behavioral Sciences. https://www.utstat.toronto.edu/~brunner/oldclass/378f16/readings/CohenPower.pdf.

[34] Craney T.A., Surles J.G. (2002). Model-Dependent Variance Inflation Factor Cutoff Values. Qual. Eng..

[35] Guo S., Kaminga A.C., Xiong J. (2021). Depression and Coping Styles of College Students in China During COVID-19 Pandemic: A Systemic Review and Meta-Analysis. Front. Public Health.

[36] Chang J.-J., Ji Y., Li Y.-H., Pan H.-F., Su P.-Y. (2021). Prevalence of anxiety symptom and depressive symptom among college students during COVID-19 pandemic: A meta-analysis. J. Affect. Disord..

[37] Pierce M., Hope H., Ford T., Hatch S., Hotopf M., John A., Kontopantelis E., Webb R., Wessely S., McManus S. (2020). Mental health before and during the COVID-19 pandemic: A longitudinal probability sample survey of the UK population. Lancet Psychiatry.

[38] Koopmans G.T., Lamers L.M. (2007). Gender and health care utilization: The role of mental distress and help-seeking propensity. Soc. Sci. Med..

[39] Barsky A.J., Peekna H.M., Borus J.F. (2001). Somatic symptom reporting in women and men. J. Gen. Intern. Med..

[40] İrer B., Çelikhisar A., Çelikhisar H., Bozkurt O., Demir Ö. (2018). Evaluation of Sexual Dysfunction, Lower Urinary Tract Symptoms and Quality of Life in Men With Obstructive Sleep Apnea Syndrome and the Efficacy of Continuous Positive Airway Pressure Therapy. Urology.

[41] Tyagi S., Chancellor M.B. (2023). Nocturnal polyuria and nocturia. Int. Urol. Nephrol..

[42] Endeshaw Y.W., Johnson T.M., Kutner M.H., Ouslander J.G., Bliwise D.L. (2004). Sleep-Disordered Breathing and Nocturia in Older Adults. J. Am. Geriatr. Soc..

[43] Bai J., Gu L., Chen Y., Liu X., Yang J., Li M., Dong X., Yang S., Huang B., Wang T. (2022). Evaluation of psychological stress, cortisol awakening response, and heart rate variability in patients with chronic prostatitis/chronic pelvic pain syndrome complicated by lower urinary tract symptoms and erectile dysfunction. Front. Psychol..

[44] Ninomiya S., Kawahara T., Tsutsumi S., Ito H., Makiyama K., Uemura H. Lower urinary tract symptoms are elevated with depression in Japanese women. Low. Urin. Tract Symptoms.

[45] Zuluaga L., Caicedo J.I., Mogollón M.P., Santander J., Bravo-Balado A., Trujillo C.G., Ritter C.D., Rondón M., Plata M. (2023). Anxiety and depression in association with lower urinary tract symptoms: Results from the COBaLT study. World J. Urol..

[46] Coyne K.S., Kvasz M., Ireland A.M., Milsom I., Kopp Z.S., Chapple C.R. (2012). Urinary Incontinence and its Relationship to Mental Health and Health-Related Quality of Life in Men and Women in Sweden, the United Kingdom, and the United States. Eur. Urol..

[47] Plata M., Bravo-Balado A., Robledo D., Trujillo C.G., Caicedo J.I., Cataño J.G., Arenas J., Rondón M., Londoño D. (2019). Prevalence of lower urinary tract symptoms and overactive bladder in men and women over 18 years old: The Colombian overactive bladder and lower urinary tract symptoms (COBaLT) study. Neurourol. Urodyn..

